# The Unforeseen Connection: A Rare Case of Salpingo-Sigmoidal Fistula Secondary to Ulcerative Colitis

**DOI:** 10.7759/cureus.84186

**Published:** 2025-05-15

**Authors:** Aagna Patel, Hamerton Jeanty

**Affiliations:** 1 Department of Medicine, Lake Erie College of Osteopathic Medicine, Erie, USA; 2 Department of General Medicine, Mohawk Valley Health System - St. Luke's Campus, Utica, USA

**Keywords:** abdominal wall abscess, crohn's disease, inflammatory bowel disease, salpingo-sigmoidal fistula, ulcerative colitis

## Abstract

Ulcerative colitis (UC) is a chronic, relapsing inflammatory bowel disease characterized by continuous mucosal inflammation confined to the colon and rectum. Although fistulizing complications are a hallmark of Crohn’s disease, they are exceedingly rare in UC, which typically involves only the mucosal layer. In these rare instances, however, prolonged and severe mucosal inflammation in UC may lead to deeper tissue injury and the development of transmural complications such as fistulas. This report details a case of a 74-year-old female patient, diagnosed with UC two years ago, who presented to our hospital with abdominal pain, mucoid diarrhea, and a lower left quadrant abdominal abscess. After CT-guided drainage of the abscess, a fistulogram confirmed a salpingo-sigmoidal fistula, with contrast outlining a tract from the sigmoid colon to the left fallopian tube and extending into the endometrial cavity. Due to the infrequency of fistula formation in UC, such a presentation can confuse the diagnosis as it does not conform to the classical pattern of UC. Therefore, this case serves to expand our understanding of the disease process and protect against a premature conclusion that would cause UC to be dismissed as a viable diagnosis when fistula formation is seen on initial presentation.

## Introduction

Ulcerative colitis (UC) is a chronic, relapsing-remitting inflammatory bowel disease (IBD) that primarily affects the colonic mucosa. Unlike Crohn’s disease (CD), which may involve any segment of the gastrointestinal tract with transmural inflammation, UC is typically limited to the mucosal layer of the colon and progresses in a continuous fashion from the rectum proximally [1]. Common clinical manifestations include bloody diarrhea, abdominal pain, urgency, and tenesmus, with patients frequently experiencing systemic symptoms such as fatigue and anemia [2]. Although the precise etiology of UC remains unclear, current evidence suggests that its pathogenesis involves a multifactorial interplay between genetic susceptibility, immune dysregulation, environmental influences, and alterations in the gut microbiome [3].

Fistula formation is a well-established complication of CD, arising from its transmural inflammatory nature. By contrast, fistulizing disease is exceedingly rare in UC, owing to its confinement to the mucosal layer. Nonetheless, in cases of long-standing, severe, or medically refractory UC, persistent inflammation may extend beyond the mucosa, predisposing to complications such as abscess formation and, in rare instances, fistula development [4]. These atypical complications are diagnostically challenging, particularly when they involve uncommon anatomical locations or present without genitourinary symptoms. Timely recognition often hinges on a high index of suspicion and the use of appropriate imaging modalities. Here, we present a rare case of a salpingo-sigmoidal fistula arising as a complication of chronic UC.

## Case presentation

A 74-year-old postmenopausal Caucasian woman presented to our hospital after outpatient imaging demonstrated redevelopment of an abscess with questionable fistula and complaints of left lower abdominal pain and mucoid diarrhea for one week. On review of systems, she denied having fever, chest pain, changes in bladder or bowel habits, passing gas through the vagina, or experiencing vaginal bleeding, discharge, itching, or odor. She noticed a soft, fluctuant mass in the lower left quadrant (LLQ) of her abdomen three days before her visit. Her medical history included multiple comorbidities, including hyperlipidemia, hypertension, and chronic apixaban use for pulmonary embolism and recurrent deep vein thromboses. Two years before this visit, she was diagnosed with UC after a colonoscopy showed moderate disease, for which she was being treated with infliximab following failure on vedolizumab. Her family history included uterine cancer in her mother and sister, and she was a lifetime nonsmoker. She was previously admitted to our hospital a month prior for an abdominal wall abscess, which was managed with surgical consultation and image-guided drainage performed by interventional radiology due to its complex loculation.

**Figure 1:**
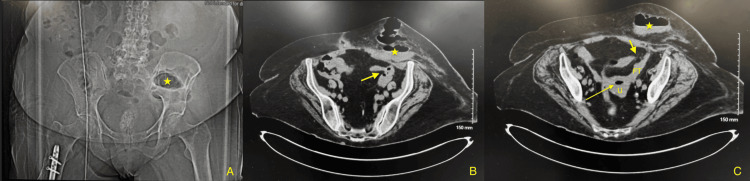
(A) Subcutaneous fluid/gas collection in the LLQ (star). (B-C) Possibility of a fistula between the colon and adnexal region (short arrow) with gas in the endometrial canal (long arrow) LLQ: Lower left quadrant

On initial admission, she was afebrile with a temperature of 98.5°F, not in acute distress, and had stable vital signs, including a heart rate of 82 bpm, respiratory rate of 20 breaths per minute, blood pressure of 121/70 mmHg, and oxygen saturation of 98% on room air. During the physical examination, a soft, tender, erythematous, tennis ball-sized fluctuating mass in the LLQ of her abdomen was palpated. No exudative material was observed during the exam. Laboratory investigations revealed microcytic anemia, leukocytosis with left-shift neutrophilia, and elevated ESR and CRP (Table 1). Blood cultures grew *Staphylococcus warneri*, which was thought to be a contaminant. A computerized tomography (CT) scan of the abdomen and pelvis showed a 7.5 cm x 4 cm x 8 cm subcutaneous fluid/gas collection in the LLQ and the possibility of a fistula between the colon and adnexal region with gas in the endometrial canal (Figures 1A-1C). General Surgery and OBGYN services were consulted, and the patient was started on empirical antibiotic coverage with intravenous piperacillin-tazobactam. She was then admitted for CT-guided drainage of the abscess.

**Table 1:** Laboratory data for the duration of the patient's hospital stay RBC: Red blood cell; MCV: Mean corpuscular volume; MCHC: Mean corpuscular hemoglobin concentration; RDW: Red cell distribution width; WBC: White blood cell; ESR: Erythrocyte sedimentation rate; CRP: C-reactive protein

Lab parameters	At diagnosis	Post-I&D	Hospital day 15	At discharge	Normal range
RBC (x 1 Mil/uL)	3.30	3.02	2.59	2.66	4.20-5.40
Hemoglobin (g/dL)	9.1	8.4	7.3	7.4	12.0-16.0
Hematocrit (%)	29.0	26.5	22.8	24.1	37.0-47.0
MCV(fL)	87.9	87.7	88	90.6	81.0-99.0
MCHC (g/dL)	31.4	31.7	32	30.7	32.2-37.0
Platelet (x 1,000/uL)	360	397	78	265	130-400
RDW (%)	16.9	16.9	15.5	16.4	11.5-14.5
WBC count (x 1,000/ uL)	15.29	7.07	3.76	4.70	4.80-10.00
Segmented neutrophils (%)	87.5	56.7	45.7	53.9	40-74
Eosinophils (%)	0.00	4.5	5.3	2.8	0-7
ESR (mm/hr)	30	-	-	-	0-29
CRP (mg/L)	-	255.00	23.30	-	0.00-3.00

**Figure 2:**
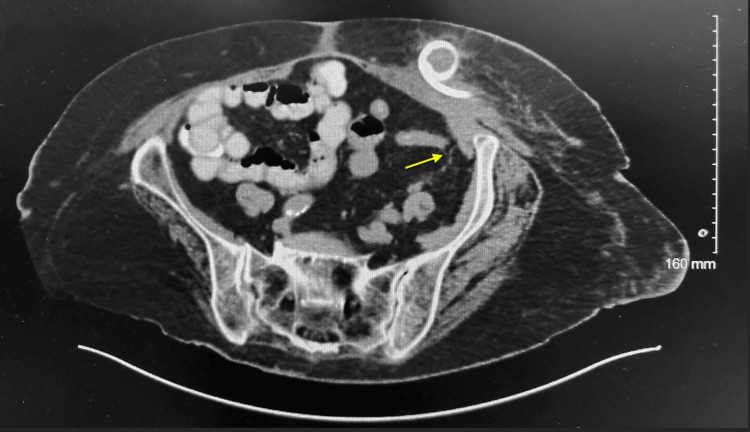
CT scan showing closely opposed bowel in the LLQ raising suspicion for an enterocutaneous fistula (arrow) LLQ: Lower left quadrant

Interventional Radiology (IR) successfully drained 70 mL of purulent fluid/gas from a large abscess in the LLQ abdominal wall. A Bellovac drain was left in place post-procedure. A follow-up CT scan performed two days later revealed no significant residual collection. However, the scan also showed closely opposed bowel in the LLQ, which raised suspicion for an enterocutaneous fistula that could not be ruled out during the abscess drainage (Figure 2). To confirm or rule out the fistula, the patient was scheduled for an LLQ fistulogram. Based on recommendations from Infectious Diseases, given her high risk, the patient was maintained on intravenous piperacillin-tazobactam and oral linezolid after the drainage. Cultures from the drained abdominal wall fluid grew *Escherichia coli*, *Enterococcus faecalis*, and *Streptococcus anginosus*, all of which were sensitive to the initial empiric therapy, thereby supporting the choice of broad-spectrum coverage. Additionally, a stool Clostridium difficile assay was conducted due to her ongoing diarrhea, which returned negative.

**Figure 3:**
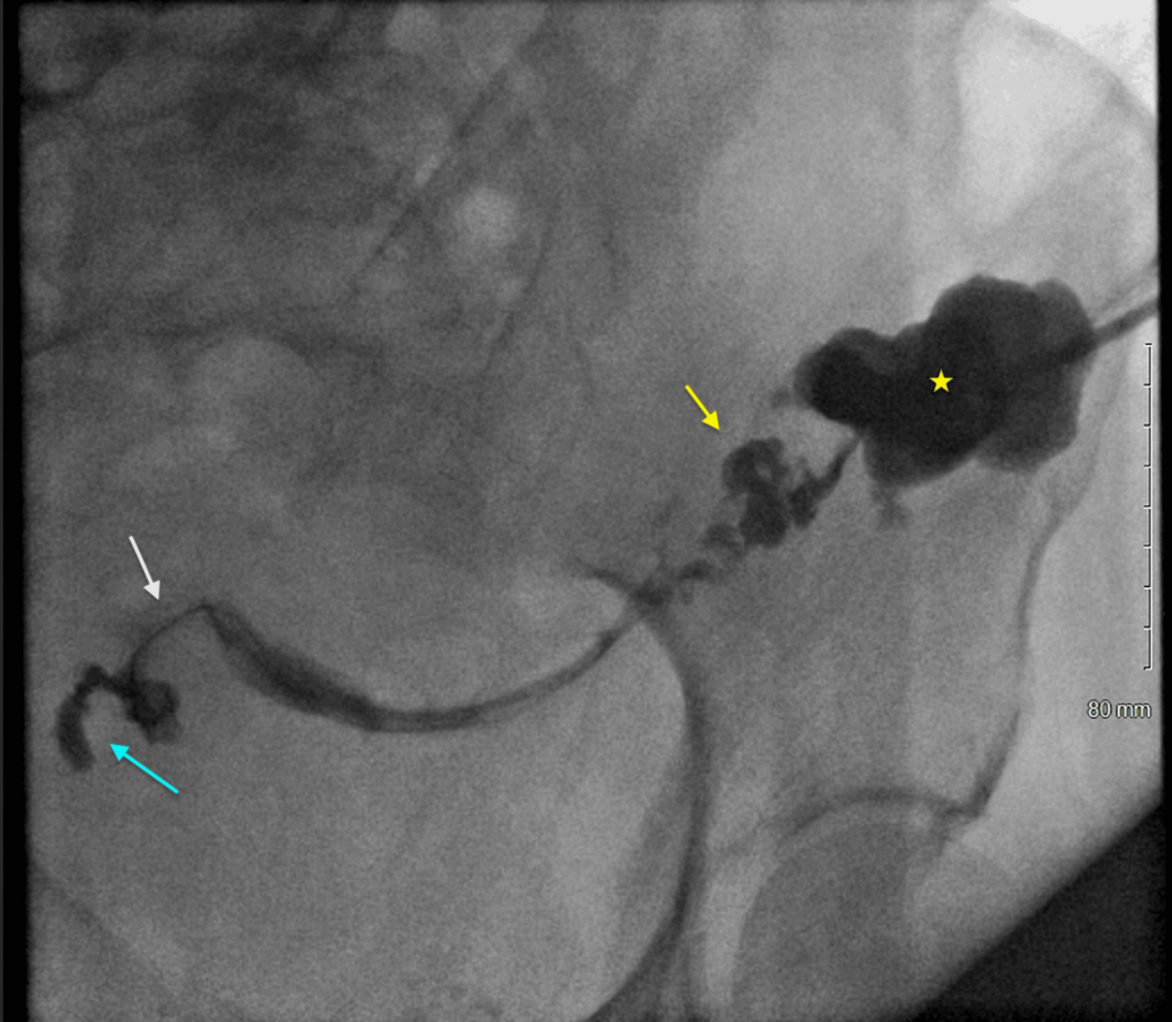
LLQ fistulogram demonstrating collapsed abscess cavity (star) and fistula communication (white arrow) with subjacent sigmoid colon (yellow arrow) and left fallopian tube (blue arrow) LLQ: Lower left quadrant

The patient remained hemodynamically stable post-percutaneous drainage of the abscess and demonstrated clinical improvement. Her white blood cell count normalized, and C-reactive protein levels declined (Table 1). An LLQ fistulogram was performed by IR two days after the drainage which revealed collapse of the abscess cavity and fistula communication with the subjacent sigmoid colon and left fallopian tube. The contrast extended through the left fallopian tube to the endometrial cavity (Figure 3). Based on these findings, along with the clinical improvement and minimal residual output, the drain was removed three days after the fistulogram. Although surgical intervention was necessary, the patient's extensive and complex medical history (including a family history of uterine cancer in her mother and sister, thickened endometrial lining from a prior pelvic ultrasound, and severe, refractory UC) posed a high risk for our hospital. Therefore, both Surgery and OBGYN recommended transfer to a tertiary center with gynecological oncologists and colorectal specialists.

Although the patient's initial post-interventional course was uneventful, she developed thrombocytopenia, worsening anemia, and hematochezia while waiting for transfer (Table 1). Hematology and Gastroenterology (GI) were consulted, and the patient received 1 unit of PRBCs. Further workup, including heparin-induced thrombocytopenia antibody, coagulation studies, and fibrinogen, was unremarkable. Stool culture, IBD panel, and Celiac panel were negative. According to Hematology, the patient's thrombocytopenia was likely secondary to prolonged linezolid treatment (three weeks). Per GI, her worsening diarrhea was likely a combination of her underlying UC exacerbated by heavy antibiotic use. Oral linezolid was discontinued, and the patient was started on cholestyramine and budesonide.

The patient’s clinical and hemodynamic status remained stable for the remainder of her hospitalization. After a multidisciplinary discussion and shared decision-making process, including a detailed explanation of the potential risks and benefits of continued inpatient management versus outpatient follow-up, the patient elected to pursue outpatient care rather than transfer to a tertiary care center. She was discharged on hospital day 20 with explicit instructions to arrange follow-up within one week with a colorectal surgeon, gynecologist, and primary care physician. In addition, referral to a gynecologic oncologist was recommended to further evaluate and guide management of her condition.

## Discussion

Fistulas are a hallmark complication of CD, arising from transmural inflammation that permits abnormal connections between the gastrointestinal tract and adjacent structures. UC, by contrast, is generally confined to the mucosa and rarely involves the deeper layers required for fistula formation. However, chronic, severe, or refractory UC can occasionally produce complications more typical of CD, including fistulas, particularly when compounded by secondary infections, tissue ischemia, or procedural trauma [1-3].

Fistulas involving the female reproductive tract, especially between the colon and fallopian tubes, are exceedingly rare. Tuboenteric or colosalpingeal fistulas have been described in fewer than 50 cases, and bilateral salpingeal transit of contrast has only been reported twice globally [4-6]. These unusual communications often arise in the context of diverticulitis, pelvic inflammatory disease, prior surgery, or radiation [4,7]. In our case, the patient had none of these common risk factors. Instead, her history of thickened endometrial lining, recent abdominal wall abscess, and ongoing UC-related inflammation likely promoted transmural inflammation and aberrant tissue remodeling, thereby creating a permissive environment for fistula formation. Moreover, our patient presented with subacute left lower quadrant abdominal pain and mucoid diarrhea, without any of the hallmark genitourinary or gynecological symptoms (malodorous or purulent vaginal discharge, fecaluria, or passage of gas per vaginam) often associated with fistulas of the female reproductive tract [4-9]. In most published cases, such symptoms are the presenting complaint and prompt further imaging or surgical exploration. The absence of these features in our patient delayed consideration of a reproductive tract fistula and underscores the diagnostic challenges in such atypical presentations.

This symptomatically silent presentation necessitated advanced imaging modalities to arrive at a definitive diagnosis, which was ultimately made using a combination of abdominal and pelvic CT imaging, as well as a left lower quadrant fistulogram. The fistulogram delineated a fistulous connection between the sigmoid colon, left fallopian tube, and the endometrial cavity. This radiographic demonstration of direct contrast passage through the fallopian tube and into the uterus is extremely rare and highlights the value of targeted imaging in complex IBD cases. While enterovesical, rectovaginal, and enterocutaneous fistulas are well documented in IBD, colosalpingeal fistulas remain virtually undocumented in the context of UC, adding to the clinical uniqueness of this case [8,10].

Following this rare radiologic confirmation, management decisions had to balance the complexity of the anatomical findings with the patient’s overall clinical risk profile. Surgical management, including en bloc resection of the involved bowel and adnexal structures, is the standard approach in most reported cases [4-9]. Only one case of spontaneous closure has been described in the literature [11]. In our patient’s case, surgical intervention was deemed high risk due to her extensive comorbidities, chronic anticoagulation, ongoing immunosuppressive therapy, and personal preference to avoid tertiary referral. Therefore, a multidisciplinary outpatient management strategy was adopted, including close follow-up with colorectal surgery, gynecology, and gynecologic oncology.

This case broadens the spectrum of UC-associated complications and demonstrates that even anatomically extensive fistulizing disease may present subclinically. It emphasizes the importance of maintaining a high index of suspicion for rare fistulous complications in longstanding UC, particularly when conventional risk factors are absent, and underscores the need to individualize care in complex medical contexts.

## Conclusions

This case underscores the potential for severe and atypical complications in ulcerative colitis, including rare instances of internal fistula formation traditionally associated with CD. We report a case of refractory ulcerative colitis complicated by a salpingo-sigmoidal fistula extending into the endometrial cavity. To our knowledge, this represents the first reported instance of such a fistula with this specific anatomical involvement and symptomatology. Clinicians should maintain a high index of suspicion for unusual complications in patients with longstanding or treatment-resistant ulcerative colitis and avoid premature closure during diagnostic evaluation. Additionally, we acknowledge the significant physical and psychological burden this complication has imposed on our patient. By sharing this case, we aim to raise awareness of such rare presentations to inform clinical vigilance and ultimately improve patient outcomes.
